# Epidemiologic and Genomic Analysis of the Severe Acute Respiratory Syndrome Coronavirus 2 Epidemic in the Nebraska Region of the United States, March 2020–2021

**DOI:** 10.3389/fmicb.2022.878342

**Published:** 2022-05-18

**Authors:** Jacob A. Siedlik, Cynthia J. Watson, Morgan A. Raine, Anne V. Cheng, Richard V. Goering, Holly A. F. Stessman, Michael Belshan

**Affiliations:** ^1^Department of Exercise Science and Pre-Health Professions, Creighton University, Omaha, NE, United States; ^2^Department of Medical Microbiology and Immunology, Creighton University School of Medicine, Omaha, NE, United States; ^3^Department of Pharmacology and Neuroscience, Creighton University School of Medicine, Omaha, NE, United States

**Keywords:** COVID-19, sequencing, genetic variation, viral evolution, epidemiology, SARS-CoV-2

## Abstract

COVID-19 emerged at varying intervals in different regions of the United States in 2020. This report details the epidemiologic and genetic evolution of Severe acute respiratory syndrome coronavirus 2 (SARS-CoV-2) during the first year of the epidemic in the state of Nebraska using data collected from the Creighton Catholic Health Initiatives (CHI) health system. Statistical modelling identified age, gender, and previous history of diabetes and/or stroke as significant risk factors associated with mortality in COVID-19 patients. In parallel, the viral genomes of over 1,000 samples were sequenced. The overall rate of viral variation in the population was 0.07 mutations/day. Genetically, the first 9 months of the outbreak, which include the initial outbreak, a small surge in August and a major outbreak in November 2020 were primarily characterized by B.1. lineage viruses. In early 2021, the United Kingdom variant (B.1.1.7 or alpha) quickly became the dominant variant. Notably, surveillance of non-consensus variants detected B.1.1.7 defining mutations months earlier in Fall 2020. This work provides insights into the regional variance and evolution of SARS-CoV-2 in the Nebraska region during the first year of the pandemic.

## Introduction

**Figure 1:**
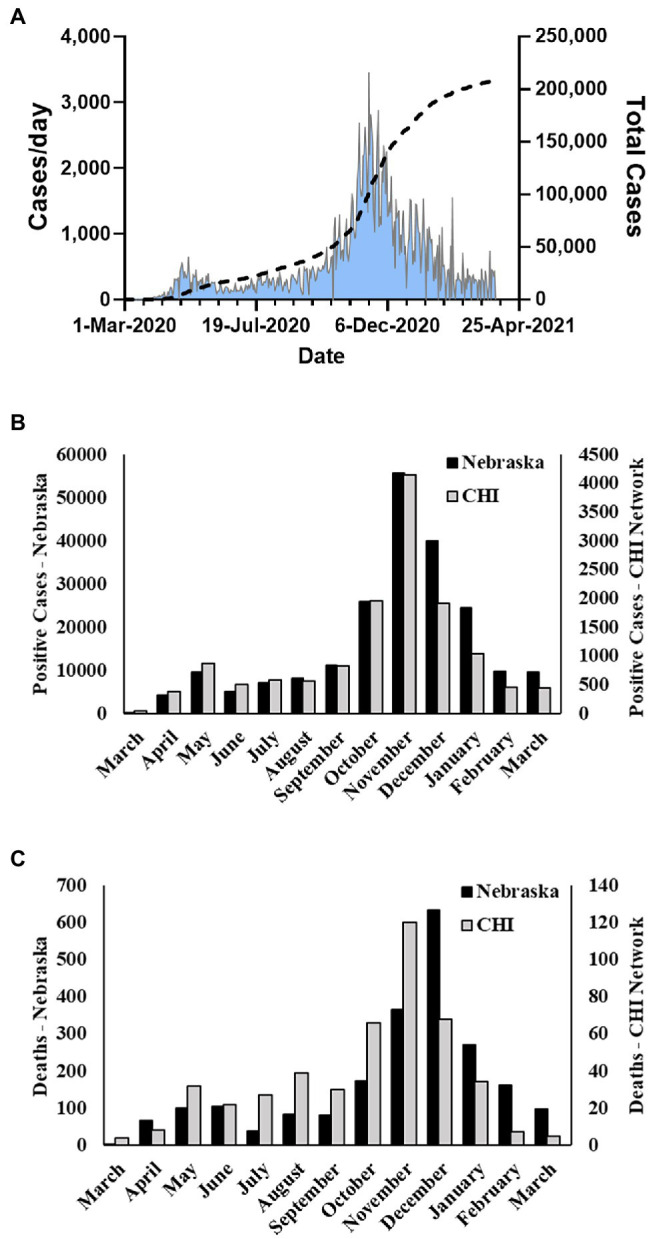
Nebraska COVID-19 cases and fatalities March 2020–March 2021. **(A)** Cases per day and total cases in Nebraska during study period as reported by NE DHHS. **(B)** Number of monthly positive cases reported by NE DHHS and number of monthly cases in the Catholic Health Initiatives (CHI) dataset used in this study. **(C)** Number of monthly fatalities due to COVID-19 in Nebraska reported by the CDC and number of monthly fatalities in the CHI dataset used in this study.

Severe acute respiratory syndrome coronavirus 2 (SARS-CoV-2), the etiologic agent of COVID-19 respiratory disease (Holshue et al., 2020; Zhu et al., 2020), is a member of the β coronavirus family and genetically related to SARS-CoV and SARS-related bat CoVs (Chan et al., 2020; Lu et al., 2020). Initially isolated in China, the outbreak spread and infected greater than 82 million persons worldwide in 2020. The United States (US) accounted for approximately 20 million of those infections. The first COVID-19 case in the United States was detected in Washington State on January 20, 2020. The virus quickly spread across the country, and the first case in the state of Nebraska was identified on March 7, 2020 in a person returning from England. Subsequently, Nebraska experienced an initial spike in cases in early to mid-May, a second milder wave in late July and August, and its largest growth in cases in mid-October through November 2020 (Figure 1A). For the year of 2020, over 170,000 cases of COVID-19 in Nebraska were reported to the CDC.

Nebraska is a land-locked state in the central region of the United States. It is ranked 37th in population of US states and home to over 1.9 million people. The state is principally rural, with four major urban centers. The largest population center is the Omaha, NE-Council Bluffs, IA metro region with a population of approximately 1.06 million. The next three most populous cities are Lincoln (~284,000), Grand Island (~51,000), and Hastings (~25,000). Notably, the Omaha area is a major ground transportation hub as it sits at the intersection of two major trucking interstate highways-I-80 and I-29. Nebraska is a unique population for several reasons, including that the state was never under any mandated stay-at-home orders, is geographically isolated, and is less densely populated than costal states. Nebraska has suffered two distinct types of outbreaks—the typical urban outbreaks associated with higher population density and “micro-industrial” outbreaks, typified by outbreaks in industrial centers such as meat-packing plants.

In collaboration with the Creighton University School of Medicine clinical partner, Catholic Health Initiatives (CHI Health), our team initiated a surveillance of COVID-19 cases utilizing the CHI Health network with the goal of characterizing patient demographic information as well as the evolution, transmission, and spread of SARS-CoV-2 in the greater Nebraska region. CHI Health is a regional network in Omaha, NE consisting of 14 hospitals, two stand-alone behavioral health facilities, and more than 150 employed physician practice locations that care for more than 1 million patients per year in Nebraska and southwestern Iowa. Epidemiologic data were extracted from the electronic medical records of over 14,000 patients, and over 1,000 samples were sequenced to assess genetic evolution and intra-host variation. Integration of these data allowed us to construct the emergence history of SARS-CoV-2 in the region, characterize the largest outbreak in November 2020, and observe the emergence of mutations associated with the United Kingdom variant that produced a second large outbreak in the state in Spring 2021.

## Materials and Methods

### Patient Samples and Viral RNA Extraction

Discarded nasopharyngeal swab samples from COVID-19 positive tests were collected from the Creighton CHI Health Clinical laboratory under Creighton IRB protocol #2001144 (initial version approved September 30, 2020). The Medical Record Number associated with each sample was recorded for retrospective data analysis; then, each sample was assigned a unique, deidentified sample code. Clinical samples were stored at −80°C until used. Viral RNA (vRNA) was isolated from 250 μl of nasal swab samples using a Maxwell® RSC Instrument (Promega, Madison, WI, United States) in the Creighton University Biorepository and Tissue Processing Core Laboratory. Samples were eluted in 50 μl of Buffer AVE (Qiagen, Germantown, MD, United States).

### Data

The CHI Health network includes region data from hospitals, care facilities, primary care, and specialist providers in the greater Omaha metro area. Clinical data were extracted from the EPIC electronic health record for all COVID-19 positive patients by the EPIC Clarity Reporting Team at Catholic Health Initiatives. A set of biometric data were obtained for each patient, including age, gender, ethnicity, race, weight, height, and body mass index (BMI). The patient class and type of encounter were extracted; for hospital encounters, the length of stay was obtained, as well as the patient outcome status (alive/deceased). The set of patient history, and pre-existing conditions or risk factors retrieved included: smoking status, long-term use of steroids (including non-steroidal anti-inflammatories, inhaled, or systemic steroids), immunosuppressive therapy, testosterone or hormone replacement therapy, allergies/asthma, cardiovascular disease, COPD, diabetes, stroke, blot clot, HIV, and pneumonia. Diagnoses were retrieved by IDC-10 codes.

### SARS-CoV-2 Genome Sequencing

Two methods were used to prepare samples for genomic sequencing. In either case, 96 samples were prepared in parallel for each sequencing run. Prior to March 2021, samples were prepared using the methods outlined by Deng et al. (2020). Briefly, 10 μl of vRNA was used in initial cDNA synthesis reactions using LunaScript Super mix (New England Biolabs, Ipswich, MA, United States). Sequencing amplicons were generated using the ARTIC nCoV-2019 v2 Panel (Integrated DNA Technologies, Iowa City, IA, United States) in two separate pools as previously described (Deng et al., 2020). Pool one and two samples were combined, cleaned up using 0.8X AMPure XP beads (Beckman Coulter, Brea, CA, United States), diluted 1:10 in nuclease-free water, and quantified using a Qubit dsDNA HS protocol (Invitrogen, Carlsbad, CA, United States). Samples were then diluted to 1 ng/μl in water and 2 ng were used for Nextera XT DNA library preparation (Illumina Inc., San Diego, CA, United States). Nextera tagmentation was followed by 18 cycles of amplification using unique i7 and i5 barcodes for each sample. After a final 0.7X AMPure XP bead clean-up, 48–96 samples were combined for sequencing on a MiSeq (Illumina) using a 2 × 251 cycle protocol. For runs after March 2021, cDNA was prepared using the NEBNext ARTIC SARS-CoV-2 Library Prep Kit (V3) as directed by the manufacturer (New England BioLabs). Briefly, 8 μl of vRNA was used in the initial cDNA synthesis reactions, and amplicons were synthesized using the NEBNext ARTIC v3 SARS-CoV-2 Primer Mix in two separate primer pools. Pools 1 and 2 were combined and purified with AMPure beads prior to end prep reaction and adaptor ligation. Samples were then purified using AMPure beads a second time prior to barcode addition. Samples were combined for a final purification prior to sequencing on a MiSeq (Illumina) using a 2 × 251 cycle protocol.

### Sequence Analysis

Samples were demultiplexed using Basespace (Illumina) and downloaded as paired (Read 1 and Read 2) FASTQ files. Paired reads were aligned to the Wuhan-Hu-1 consensus (Genbank #MN908947.3) using bwa v0.7.17-r1188 (Li and Durbin, 2009) and converted to BAM files using Samtools v1.10 (Li et al., 2009). In the aligned file, primer regions and low-quality bases are soft clipped off based on a primer position BED file using iVar v1.3.1 (Grubaugh et al., 2019). Variants were called using Samtools mpileup, and a consensus was generated using iVar including variant bases with a read depth greater than 20X and a frequency greater than 60%. Coverages for all consensus FASTA sequences were calculated, and any sequence with <70% total genome coverage was removed from the downstream analysis. Lineage assignments were made using consensus sequences through the Pangolin COVID-19 Lineage Assessor website.^1^ Variants were defined as an allele frequency deviating from the reference genome at greater than 10% of reads. The non-reference allele count (DV) and sequencing depth (DP) were extracted for each sample using Samtools to calculate variant frequencies. Variants of interest were visualized using the Integrated Genomics Viewer (Robinson et al., 2011). Sequence data can be accessed through the GISAID database.^2^ GISAID accession numbers are provided in supplementary materials.

### Statistical Analysis

We retrospectively reviewed clinical data from 13,727 COVID-19 positive events in the CHI Health network for the state of Nebraska during the time frame from March 19, 2020 to March 31, 2021. Continuous variables are presented as means ± SD, and independent samples *t* tests were used to compare difference between groups when necessary. Categorical variables are shown as frequency with percentages and analyzed using Pearson’s *c*^2^ tests when appropriate. The primary goal of this analysis was to determine a predictor model for mortality risk. Predictive variables of interest included pre-existing conditions known at the time of initial patient contact such as: demographic features (e.g., age, height, and sex), comorbidities (e.g., cardiovascular disease and diabetes), and history of smoking (never, current, and former). A binomial logistic regression model was utilized to determine the effect of predictor variables on patient outcomes (alive or deceased). Potential predictors were initially analyzed using univariate logistic regression models, followed by a backward stepwise multivariate logistic regression model that excluded non-significant variables identified from the univariate outputs. Odd ratios with associated 95% CI are reported for retained predictors. Parameter estimates were interpreted as statistically significant if the 95% CI did not include zero. For all analyses, *a* < 0.05. All statistical analyses were performed in R version 4.1.1 (R Development Core Team, 2021). Packages utilized for data wrangling and analysis included *dplyr*, *tidyverse*, and *pscl*. Data visualization was completed using *ggplot2*.

## Results

### SARS-CoV-2 Dynamics in Nebraska

During the period of March 19, 2020–March 31, 2021, the state of Nebraska reported 211,015 COVID-19 cases (Figure 1A). Within this same time period, there were 13,727 positive events reported across 12,690 unique patients within the Creighton University Medical Center/CHI Health system representing 6.5% of the state’s total positive cases during the first year of the COVID-19 pandemic (Figure 1B). Within our data set, we observed a case fatality rate of 3.6% (Figure 1C), which is higher than the reported rate for the state of Nebraska (1.03%). However, our data were collated from a hospital network and are likely skewed toward more severe cases. To this point, 37.8% of the observed cases in our dataset were associated with hospital visits (average duration of stay = 15.9 ± 18.4 days), whereas the reported overall hospitalization admission rate for Nebraska during this time period was 11.5%.^3^ Males were significantly underrepresented in the hospital visit data relative to females (χ^2^(1) = 24.8, *p* < 0.001), but were significantly overrepresented in the deceased population (χ^2^(1) = 31.1, *p* < 0.001) with a 4.7% case fatality rate compared to 2.8% in females (Table 1). Of note, 1,658 patient events did not contain information on patient outcome and were, therefore, not included in patient outcome calculations.

**Table 1:** Demographic characteristics of sample population.

	Population		Alive		Deceased	
	*n*	Mean ± *SD*	*n*	Mean ± *SD*	*n*	Mean ± *SD*
Age (years)	12,690	49.3 ± 21.7	10,570 (83.2%)	46.6 ± 20.3	462 (3.6%)	78.1 ± 12.6
Height (cm)		167.7 ± 14.8		167.7 ± 14.8		170.2 ± 10.4
Weight (kg)		87.2 ± 27.5		87.7 ± 27.4		86.1 ± 26.9
BMI		30.7 ± 9.6		30.9 ± 9.5		29.7 ± 8.8
Male (44.9%)						
Age (years)	5,708	48.3 ± 21.2	4,726 (82.7%)	46.8 ± 20.3	268 (4.7%)	76.8 ± 11.9
Height (cm)		174.9 ± 15.4		174.9 ± 13.3		176.3 ± 7.9
Weight (kg)		93.5 ± 28.4		93.9 ± 28.6		91.9 ± 25.5
BMI		30.1 ± 9.6		30.3 ± 10		29.4 ± 7.7
Female (55%)						
Age (years)	6,982	47.4 ± 21.1	5,844 (83.7%)	46.4 ± 20.3	194 (2.8%)	79.8 ± 13.3
Height (cm)		162 ± 11.6		162 ± 11.6		161.5 ± 6.9
Weight (kg)		82.6 ± 25.6		82.9 ± 25.4		78 ± 26.9
BMI		31.3 ± 9.6		31.3 ± 9		30.2 ± 10.1

**Figure 2:**
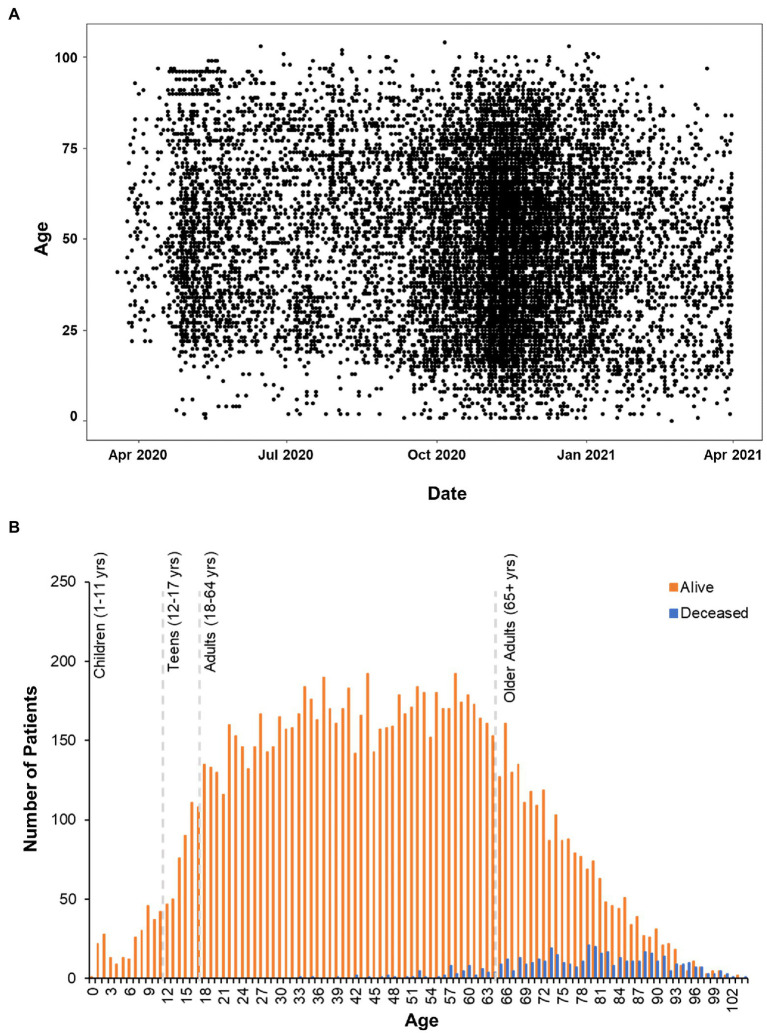
Age Distribution of patients analyzed in clinical outcomes study. **(A)** Scatterplot of positive COVID-19 test dates by age in the CHI dataset. **(B)** Histogram plot of all the patients in the data set. Survivors are plotted in orange bars and those that died with blue bars.

Positive cases spanned 0–104 years of age (Figure 2A). Deceased individuals were significantly older than survivors [*t*(759.48) = 54.43, *p* < 0.001], with an average age of 47.96 ± 20.94 years (range: 0–103 years) for the survivors compared to 78.01 ± 12.44 (range: 33–104 years) years for the deceased (Figure 2B). There were no significant differences in weight [Surviving = 87.75 ± 27.42 kg, Deceased = 86.11 ± 26.94 kg, and *t*(501.17) = 1.28, *p* = 0.2] between the surviving and deceased patients, however, BMI was modestly, but significantly decreased in the deceased population [Surviving = 30.86 ± 9.48 kg/m^2^, Deceased = 29.73 ± 8.79 kg/m^2^, and *t*(507.13) = 2.67, *p* = 0.007]. Overall, 41.9% of the patients in our data set were classified as obese (BMI ≥ 30) which is in line with the national rate of 42.4% in the United States (Warren et al., 2021), but greater than the Nebraska state average of 34% in 2020. The highest BMI values were seen in the adult age range (18–64 years: 31.7 ± 8.3 kg/m^2^) with a slight decrease observed in those 65 years and older (30.1 ± 7.2 kg/m^2^). Further investigation of the older adult population (*n* = 2,998) found that age and BMI were inversely correlated (*r* = −0.28, *p* < 0.001) indicating that as age increases in this portion of the population BMI decreased perhaps explaining the trend toward lower BMI among deceased patients.

The most prevalent comorbidities in the dataset were cardiovascular diseases (32.8%) and diabetes (13.4%) with 12.3% of the COVID-19 positive patients exhibiting both conditions (Table 2). Those patients with both cardiovascular disease and diabetes had a 55.4% hospitalization incidence (length of stay 11.6 ± 13.3 days) and an 11.7% case fatality rate. Around 13.4% of the individuals in the dataset had diabetes, which is in line with the 13% of United States adults with diabetes (CDC, 2021). The 32.8% incidence of cardiovascular disease is lower than the national average of 49.2% reported for 2018 (Virani et al., 2021).

**Table 2:** Reported comorbidity data.

Reported comorbidity	Population			Male			Female		
	Total^1^	Alive	Deceased	Total	Alive	Deceased	Total	Alive	Deceased
Allergies/Asthma	1,555 (12.3)	1,491 (95.9)	64 (4.1)	585 (4.6)	556 (95)	29 (5)	970 (7.6)	935 (96.4)	35 (3.6)
Blood clots	202 (1.6)	171 (84.7)	31 (15.3)	102 (0.8)	79 (77.5)	23 (22.5)	100 (0.8)	92 (92)	8 (8)
CVD	4,156 (32.8)	3,784 (91)	372 (9)	2,005 (15.8)	1,795 (89.5)	210 (10.5)	2,151 (17)	1,989 (92.5)	162 (7.5)
COPD	168 (1.3)	142 (84.5)	26 (15.5)	71 (0.6)	56 (78.9)	15 (21.1)	97 (0.8)	86 (88.7)	11 (11.3)
Diabetes	1,696 (13.4)	1,486 (87.6)	210 (12.4)	849 (6.7)	725 (85.4)	124 (14.6)	847 (6.7)	761 (89.8)	86 (10.2)
Diagnosed HIV	14 (0.1)	13 (92.9)	1 (7.1)	14 (0.1)	13 (92.9)	1 (7.1)	0 (0)	0 (0)	0 (0)
HRT	17 (0.1)	17 (100)	0 (0)	3 (0)	3 (100)	0 (0)	14 (0.1)	14 (100)	0 (0)
NSAIDs	12 (0.1)	12 (100)	0 (0)	6 (0)	6 (100)	0 (0)	6 (0)	6 (100)	0 (0)
Pneumonia	11 (0.1)	10 (90.9)	1 (9.1)	7 (0.1)	6 (85.7)	1 (14.3)	4 (0)	4 (100)	0 (0)
Stroke	235 (1.9)	179 (76.2)	56 (23.8)	115 (0.9)	79 (68.7)	36 (31.3)	120 (0.9)	100 (83.3)	20 (16.7)
Systemic steroids	30 (0.2)	23 (76.7)	7 (23.3)	10 (0.1)	6 (60)	4 (40)	20 (0.2)	17 (85)	3 (15)
Testosterone therapy	81 (0.6)	74 (91.4)	7 (8.6)	81 (0.6)	74 (91.4)	7 (8.6)	0 (0)	0 (0)	0 (0)

1 Data are presented as frequency (percent of sample).

**Figure 3:**
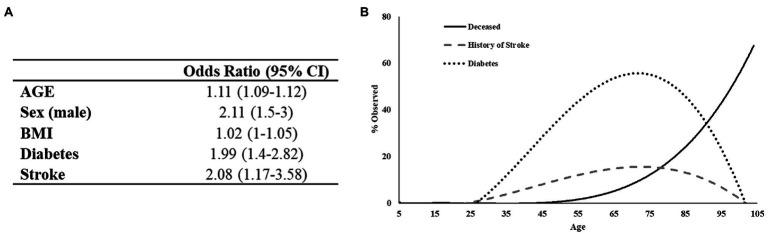
Regression analysis of preexisting conditions associated with mortality in COVID-19 positive patients. **(A)** The deceased population shows an increased tendency toward diabetes and to a lesser extent history of stroke. **(B)** Logistic regression output. Test/Train model has 88% predictive probability.

To develop a predictor model for mortality risk, we began with an investigation of variables specific to patient demographics and self-reported comorbidities. Initially, 22 variables were analyzed using univariate logistic regression models to identify factors associated with patient mortality. Twelve variables were found to be predictive of patient death in this primary analysis including: age, sex, height, BMI, smoking status, systemic steroids use, testosterone therapy, cardiovascular disease, diabetes, stroke, COPD, and history of blood clots. Subsequently, these variables were entered in a multivariate stepwise binomial logistic regression model, which identified five variables as statistically significant determinants of patient mortality (Figure 3A). This final logistic regression model indicated that a year increase in age increases the odds of death by 11% and that males are 2.11 times more likely to die from COVID-19. These data are aligned with the results of the χ^2^ analysis above. The model also indicated that patients with a history of diabetes or stroke have a 1.99 and 2.08 times, respectively greater risk of death independent of age. Thus, patients dying at the younger end of the age spectrum are disproportionately affected by either diabetes or stroke relative to those of greater age (Figure 3B). Overall, the model predicted 88% of outcomes correctly with a McFadden’s *R*^2^ = 0.33.

**Figure 4:**
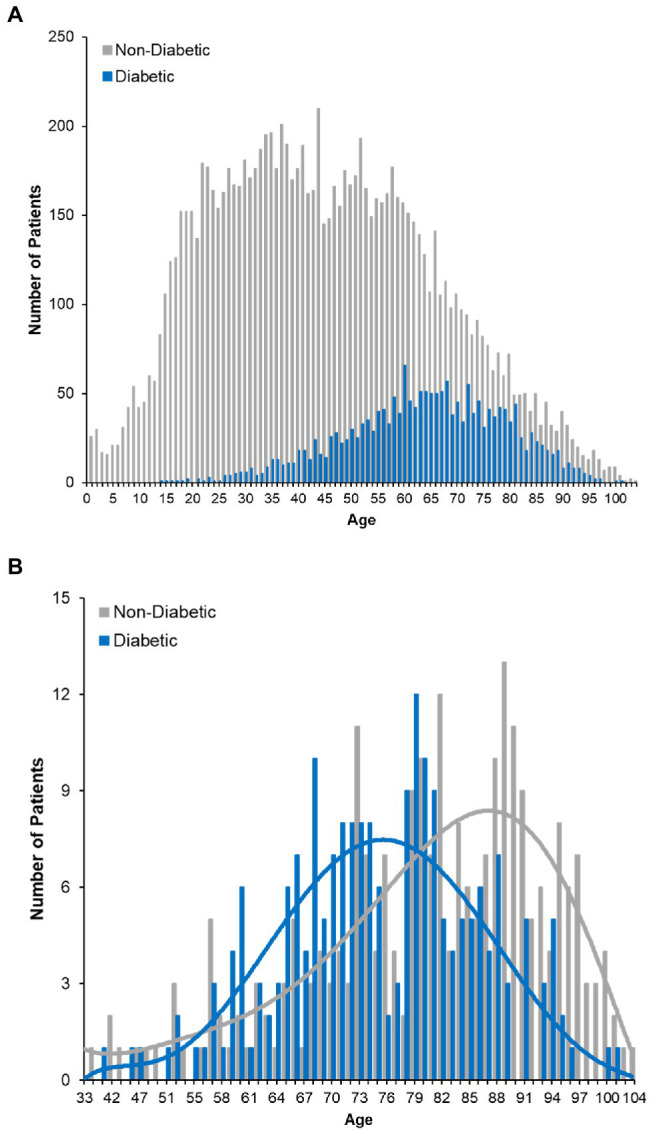
COVID-19 fatality and diabetes. **(A)** Histogram plot of the ages of diabetic COVID-19 patients in dataset. Survivors are plotted with red bars and those that died with green bars. **(B)** Age distribution of deceased patients bifurcating non-diabetic and diabetic patients.

The increased risk of death associated with diabetes was of particular interest given its high prevalence in our dataset (Figure 4A) and across the United States. Further analysis of the deceased patients revealed that the proportion of individuals with diabetes is greater than would be expected [χ^2^(1) = 332.97, *p* < 0.001]. Moreover, deceased patients with a history of diabetes were approximately 5.4 years younger than deceased patients without a diabetes diagnosis (95% CI = 3.16–7.59 years, *p* < 0.001), even though a greater number of non-diabetic patients died (252 non-diabetics vs. 210 diabetics; Figure 4B). Patients exhibiting both diabetes and a history of stroke (*n* = 114, 7.8% of the deceased population) had a 31.6% case fatality rate. These data suggest that either alone, or in combination, diabetes and stroke represent significant indicators of mortality among COVID-19 positive patients.

### Phylogenetic Analysis of SARS-CoV-2

**Figure 5:**
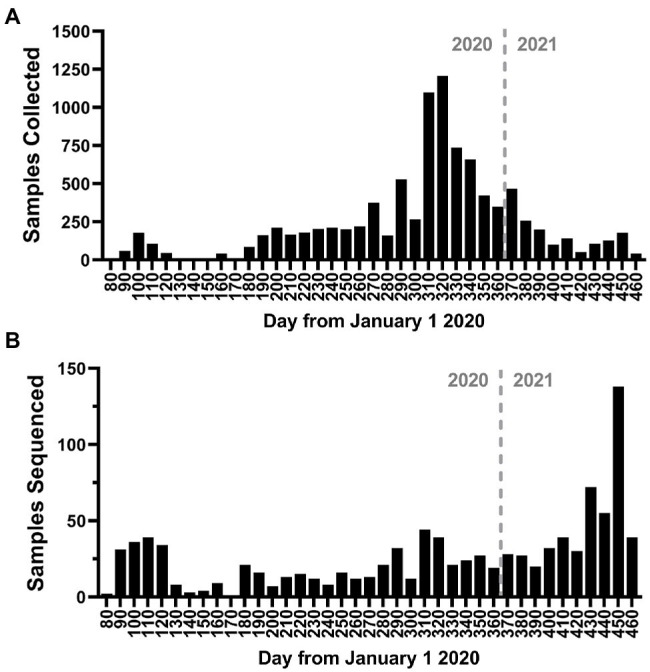
Study sampling for genome sequencing. **(A)** Histogram of the number of positive nasal-pharyngeal severe acute respiratory syndrome coronavirus 2 (SARS-CoV-2) positive samples collected during the study period. **(B)** Histogram of the number of samples sequenced during the study period. The bin depth for both histograms is 10 days.

To better understand the evolution and persistence of SARS-CoV-2 in the region throughout the period of epidemiological analysis, we sequenced the whole genomes of viruses circulating in the greater Omaha region during March 19, 2020–March 31, 2021. A total of 13,727 positive samples were reported in the CHI Health system, and a total of 9,591 samples (69.9%) were archived (Figure 5A). Importantly, the collection of samples mirrored the overall frequency of positive cases as they occurred in the state (compare Figure 5A to Figure 1A). A subset of 1,023 samples representing 7.45% of patient samples collected, were processed for whole genome sequencing using the ARTC protocol. Sequence data was processed using the iVar protocol (Grubaugh et al., 2019), and only samples with ≥70% genome coverage were used in downstream analyses. Using this cutoff, a total of 960 genomes were analyzed for this study. The temporal distribution of these samples is shown in Figure 5B. Overall, the samples were distributed throughout the year with the exception of a period of low sequencing in June 2020 due to unavailable samples as well as a period of higher sequencing in March 2021 as surveillance sequencing increased.

**Figure 6:**
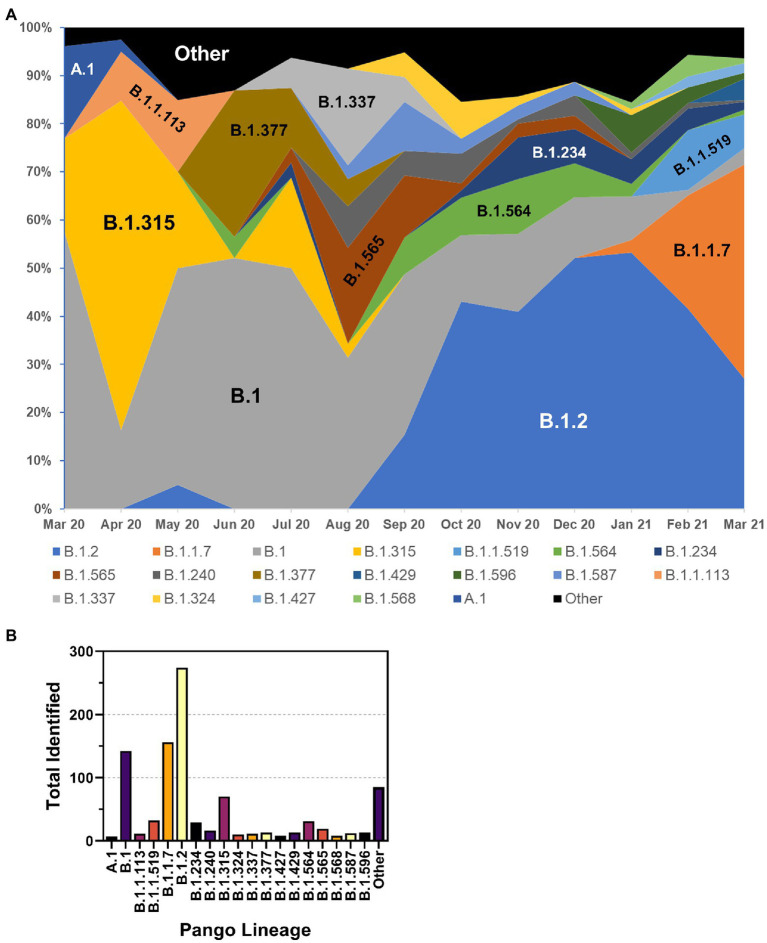
Distribution of PANGO lineages of NE samples. **(A)** Monthly frequency distribution of the PANGO lineages of the most prevalent lineages observed among sequenced samples. **(B)** Summary graph of the total number of samples observed from March 2020–March 2021 of indicated PANGO lineages.

The frequency of the most prevalent Pangolin lineages by month is shown in Figure 6A. While initially present, A.1 lineage viruses disappeared in Spring 2020. The first Nebraska peak of infections was predominated by B.1 and B.1.315 viruses, as well as B1.1.113 and B.1.377 viruses. The B.1.315 lineage is defined by the shared genomic variant A24,129G which results in a N856S substitution in Spike. The cluster arose early in the epidemic in April 2020 but subsequently disappeared. These data are consistent with local clustered epidemics, including 12 reports of outbreaks in meatpacking plants throughout the state. Indeed, during the 2020 summer months, a relative period of lower rates of infection, numerous lineages were observed at low frequency, including B.1.337 and B.1.565 viruses. B.1.2 lineage viruses began increasing in September 2020 and became the most prevalent lineage associated with the major outbreak in November 2020. B.1.2 remained prevalent into 2021. United Kingdom variant (B.1.1.7 or alpha) viruses then appeared early in the new year and supplanted B.1.2 as the dominant strain by spring 2021 (data not shown). The overall total number of samples for each major lineage is shown in Figure 6B. The most common lineage detected in the first year of the pandemic in Nebraska was B.1.2, followed by B.1.1.7 and B.1.

### Genomic Variation and Evolution

**Figure 7:**
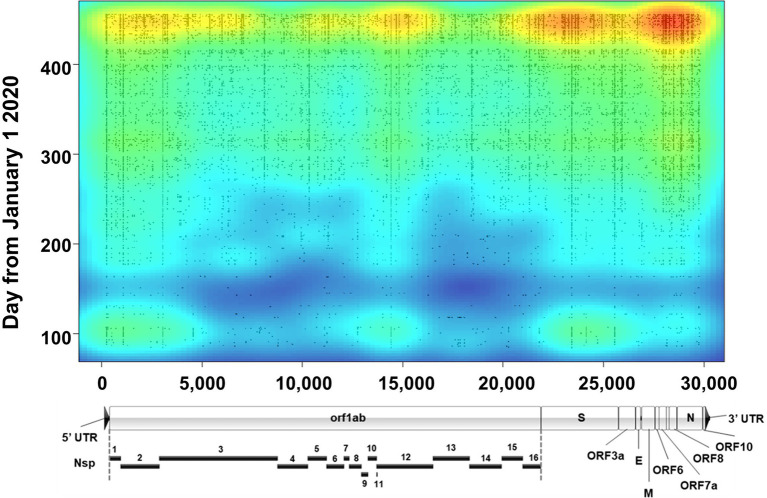
Severe acute respiratory syndrome coronavirus 2 variation. Plot shows a heat map of genomic variation relative to the Wuhan-Hu-1 reference genome. Areas of low and high variation are shown as blue and red, respectively. Plot is overlayed with individual dots that denote the locations of high-quality variants (both consensus and minor variants) identified in all sequenced samples as a function of time (*y* axis). For reference, a to-scale map of the genome is shown below with known proteins labeled. Nsp, nonstructural protein.

Lineages are defined by specific, characteristic, usually non-synonymous, mutations divergent from the original Wuhan-1 sequence (for example, B.1 is defined by three amino acid substitutions-P314L in Orf1b, D614G in Spike, and S84L in Orf8). However, assessment of the true evolution of the viruses circulating in a population necessitates identifying all mutations occurring in samples. To accomplish this, we mapped all genetic (synonymous and non-synonymous) mutations across all sequenced samples and plotted them as a function of time with an overlayed heat map (Figure 7). Several notable things can be seen with this visualization. First, although mutations occurred across the genome, there appear to be three main regions of variation. The most variable region of the genome was the 3′ end including Spike and the downstream orfs, but substantial variation was also seen at the 5′ end (nt 1–3,000, including nsp1 and the N-terminus of nsp2) and a central region in *orf1b* (nt 14,000–15,000), corresponding to the nsp12 polymerase. The occurrence of several common mutations that arose early and remained stable can be observed by continuous vertical lines. These included mutations common to the B.1 lineage viruses (ORF1b P314L, Spike D614G, and ORF8 S84L) as well as the common mutations listed in Table 3. Finally, around the time of the appearance of B.1.1.7 (January 2021, ~day 370 on plot), there was a substantial increase in the total number of mutations as seen on the heat map.

**Table 3:** Most common consensus variants.

Position	Mutation	Feature	Amino Acid	Total	Frequency	% of Samples
241	C ➔ T	3′ non-coding	N/A	928	0.999 ± 0.007	96.7%
1,059	C ➔ T	*orf1a* (nsp2)	Thr265Ile	596	0.933 ± 0.184	62.1%
3,037	C ➔ T	*orf1a* (nsp3)	*Syn.*^1^	948	0.999 ± 0.008	98.8%
14,408	C ➔ T	*orf1b* (nsp12)	Pro314Leu	950	0.999 ± 0.005	99.0%
23,403	A ➔ G	Spike	Asp614Gly	952	0.999 ± 0.007	99.0%
25,563	G ➔ T	Orf3a	Gln57His	608	0.934 ± 0.180	63.3%

1 Synonymous mutation.

**Figure 8:**
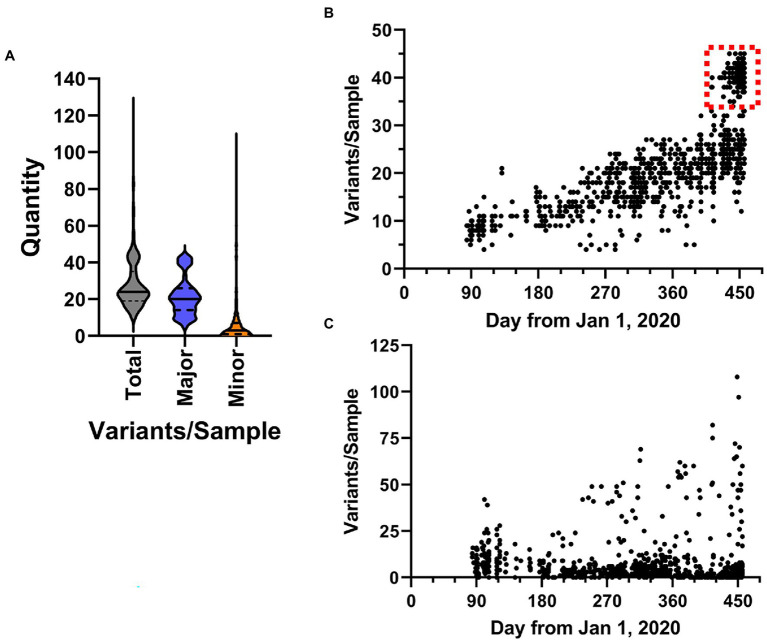
Characteristics of SARS-CoV-2 variation in samples. **(A)** Overall distribution of the number of variants identified in each sample shown as violin plots. Total includes all variants in the dataset, major and minor are defined by frequency ≥0.6 and <0.6, respectively. **(B,C)** The frequency SARS-CoV-2 variants in samples over time. Plots show the total number of major (b, frequency ≥ 0.6) and minor (c, frequency < 0.6) variants identified in samples from March 1, 2020 to March 31, 2021.

Importantly, analysis of all sequencing reads at the individual level provides the most comprehensive picture of viral variation in a patient sample. Notably, the iVar pipeline calls consensus small nucleotide polymorphisms (SNPs) as changes at a frequency of 0.6 of above. We also analyzed the outputted variant call files to identify both consensus (termed “major”) variants as well as “minor” variants, which we defined as those occurring at a frequency >0.1 and <0.6, using a minimum read depth of 200 to avoid artifacts. A violin plot of the overall number of variants identified in each sample is shown in Figure 8A. Overall, in the entire dataset, there was a mean of 28.41 ± 14.03 SNPs per sample. The majority of SNPs were major variants (74.5%). Temporal plotting of the number of major and minor variants in each sample is shown in Figures 8B,C, respectively. Consistent with continual evolution of the virus, the number of major variants increased over time. Simple linear regression produced a best fit slope of 0.07 mutations/day (2.1 mutations/month) with a *r*^2^ value of 0.598, consistent with previous observations (Alteri et al., 2021; Page et al., 2021). Also notable in the temporal plot is the appearance of a distinct cluster of samples with higher variant counts that coincided with the introduction of B.1.1.7 lineage viruses (denoted by dashed red box in Figure 8B). The plot of the minor variants identified per sample showed a constant level of minor variants present in samples, but also a distinct subpopulation of minor variants that increases over time (Figure 8C). This correlated with the linear increase in major variants over time.

**Figure 9:**
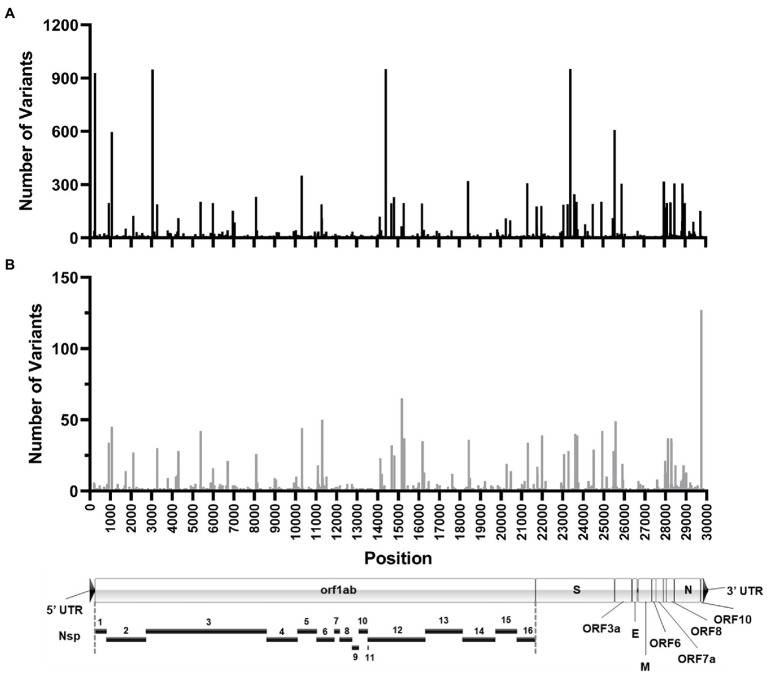
Location of SARS-CoV-2 variation identified in samples. Histograms shows the number of high-quality major **(A)** and minor **(B)** variants identified at each genomic position in all the patient samples sequenced. Position location is relative to the Wuhan-Hu-1 reference genome. **(A)** To-scale map of the genome is shown below with known proteins labeled. Nsp, nonstructural protein.

The sum total of major variants identified by genomic location is shown in Figure 9A. Within the 960 samples in the study period there were 2,076 positions across the genome that produced major variants. As temporally displayed in Figure 7, there were a small number of SNPs that were present in almost all the samples. These common major variants are listed in Table 3. There were also 1,423 positions of minor variation detected, and 671 positions with both major and minor variants detected among the samples. The distribution of the minor variants is shown in Figure 9B. Whereas the major variants clustered mostly in the three regions noted earlier, the minor variants were more regularly distributed across the genome. The most commons sites of minor variation are presented in Table 4. Notably, two of those positions were also found to have major variants in numerous samples.

**Table 4:** Most common minor variants^1^.

Position	Mutation	Feature	Amino Acid	Total^2^	Frequency^3^	% of Samples^3^
11,296	T ➔ R	orf 1a (nsp6)	Phe3690Leu	50 (61)	0.652 ± 0.315	11.56%
15,168	G ➔ A	*orf1b* (nsp12)	*Syn.*^4^	65	0.211 ± 0.026	6.78%
15,173	C ➔ A	orfb (nsp12)	Ser569STOP	60	0.136 ± 0.019	6.25%
29,737	G ➔ C	3′ non-coding	N/A	127 (24)	0.402 ± 0.228	15.73%

1 Variants identified in iVar analysis at frequency < 0.6.

2 Major variant (≥0.6) count in addition to minor frequency variants in parentheses.

3 Calculated using both major and minor variants.

4 Synonymous mutation.

**Figure 10:**
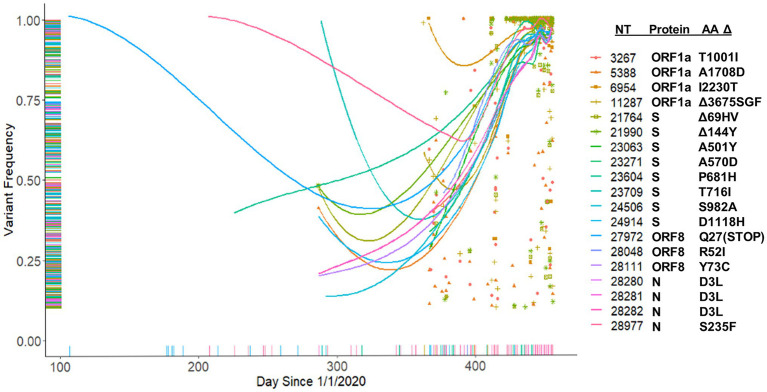
Tracking of B.1.1.7 mutations. Frequency of B.1.1.7 variants identified in the patient samples beginning from January 1, 2020.

We hypothesized that major variants necessarily arise in individuals initially as minor variants. This idea is supported by the identification of 671 sites containing both minor and consensus variants, as well as the parallel increase in consensus and a subset of minor variants (Figures 8B,C). To investigate this idea, we examined the temporal frequency of the mutations that define the United Kingdom variant, which rose to prominence in the sample set late in our sampling period (Figure 10). Almost all the mutations were observed as minor variants priors to the appearance of the B.1.1.7 lineage in Nebraska. Regression lines for each of the positions clearly show that most SNPs were first detected as low, minor frequency variants prior to becoming consensus variants in late 2020/early 2021.

## Discussion

In this study we examined patient medical records and sequenced ~1,000 SARS-CoV-2 genomes to retrospectively explore health outcomes and viral diversity and evolution during the first year of the COVID-19 outbreak in Nebraska. Nebraska’s first case was reported March 7, 2020 as a travel-related case of a patient returning sick from England. In the first month, Nebraska saw a rapid onset of over 80,000 cases. Shortly thereafter, the state announced the closure of schools and restrictions in restaurants in an attempt to limit transmission and the length of time which people spent in close proximity to one another. For the first months of the pandemic, infected individuals were hospitalized as a way to quarantine them until they tested negative. However, hospital facilities were quickly overrun, and residents were encouraged to quarantine at home if exposed to the virus. Rapid testing was made available in the first weeks of May 2020, and the first state-wide mask mandate was introduced at the end of July requiring masks to be worn in all indoor settings. Despite these interventions, the state experienced waves of increased cases including a minor outbreak in August 2020 followed by large outbreaks in September 2020, April 2021, and August 2021. Notably, the free COVID-19 testing provided by the state of Nebraska may have biased our clinical data and sequencing samples toward symptomatic cases as samples processed by the Creighton CHI Health Clinical Laboratory came primarily from health care facilities.

Much of the retrospective clinical data analysis (69.5%) consisted of either hospital contacts (*n* = 5,193) or clinical office visits (*n* = 4,352), which suggests patients were self-reporting as symptomatic and seeking out clinical testing. As evidence of this, it is not until April 24, 2020 that we saw the first positive case in someone under the age of 22 appears in this data set. Thus, our findings may be biased toward ill vs. asymptomatic individuals. Overall, our study demonstrates that mortality risk among COVID-19 positive patients is influenced by a combination of age, sex, and the presence of specific comorbidities. These findings are supported by previous reports that show males over the age of 65 years with comorbidities like diabetes are at a greater risk of severe disease and mortality (Hui et al., 2020; Williamson et al., 2020; Zheng et al., 2020; Zhou et al., 2020; Zhang et al., 2020a,b; Betti et al., 2021; Velasco-Rodriguez et al., 2021). To this point, only 22.3% of our patient sample was over the age of 65 years, but this population accounted for 84.8% of the overall deaths and was disproportionately male. For deceased patients under the age of 65, they averaged 1.7 pre-existing conditions (range = 0–4) and 57 ± 7.4 years of age. We identified eight patients under the age of 65 (55.6 ± 8.8 years) without any pre-existing conditions that died with a COVID-19 positive status representing 0.06% of our total population. Together, these data support existing research finding that older age and the certain comorbidities, specifically diabetes, are associated with an increased risk of death in COVID-19 positive patients (Hui et al., 2020).

Our sequence data provide a broad survey of the diversity and evolution of SARS-CoV-2 during the first year of the pandemic in Nebraska. Although a few A lineage viruses were detected early, these were quickly displaced by B.1 lineage viruses which predominated throughout the year. The most prevalent lineage observed in 2020 was B.1.2, which was the most frequent variant observed during the largest outbreak in November 2020. Based on the number of identified major variants per sample over time, we observed a population rate of 0.7 mutations/day, which, as expected, resulted in an overall increase in viral variation throughout the year. However, the introduction of B.1.1.7 produced a notable shift in the number of variants per sample (Figure 8B). It will be interesting to determine if the observed population rate remains steady as ongoing studies continue to track the rate of viral variation in samples during 2021 and 2022.

Simple retrospective analyses of consensus sequences, such as those available in public databases (GISAID), is of limited utility as they provide only unidimensional data. The United Kingdom variant, B.1.1.7 established itself as the dominant variant in Nebraska and the overall United States by March 2021 (Alpert et al., 2021; Washington et al., 2021). By investigating the occurrence of minor SNPs, we were able to observe the presence of all of the key mutations that define the B.1.1.7 lineage in late 2020. This is interesting given the proposal that B.1.1.7 may have arisen from a recombination event (Jackson et al., 2021). Nevertheless, these data provide evidence that there may be a benefit in monitoring minor variants. Future analyses of the emergence of SNPs comprising the Delta and Omicron variants are ongoing to validate this approach and lead to a more accurate method to predict potential new variants of interest before they arise.

## Data Availability Statement

The datasets presented in this study can be found in online repositories. The names of the repository/repositories and accession number(s) can be found at: NCBI—PRJNA690551, OM756783–OM757734.

## Ethics Statement

The studies involving human participants were reviewed and approved by Creighton University Institutional Review Board. Written informed consent for participation was not required for this study in accordance with the national legislation and the institutional requirements.

## Author Contributions

JS, CW, MR, AC, HS, and MB performed the research. JS, AC, RG, HS, and MB curated and analyzed the data. JS, CW, HS, and MB wrote the manuscript. JS, CW, RG, HS, and MB reviewed and revised the manuscript. All authors contributed to the article and approved the submitted version.

## Funding

Funding for this work was provided by the Creighton University School of Medicine, the Nebraska Department of Health and Human Services, and the State of Nebraska Tobacco Settlement LB595 from Creighton University to HS.

## Conflict of Interest

The authors declare that the research was conducted in the absence of any commercial or financial relationships that could be construed as a potential conflict of interest.

## Publisher’s Note

All claims expressed in this article are solely those of the authors and do not necessarily represent those of their affiliated organizations, or those of the publisher, the editors and the reviewers. Any product that may be evaluated in this article, or claim that may be made by its manufacturer, is not guaranteed or endorsed by the publisher.

## Footnotes

^1^ https://pangolin.cog-uk.io/

^2^ https://www.gisaid.org/

^3^ healthdata.gov
